# MiR-194 Regulates Chondrogenic Differentiation of Human Adipose-Derived Stem Cells by Targeting Sox5

**DOI:** 10.1371/journal.pone.0031861

**Published:** 2012-03-01

**Authors:** Jun Xu, Yan Kang, Wei-ming Liao, Ling Yu

**Affiliations:** 1 Department of Orthopedics, The First Affiliated Hospital, Sun Yat-sen University, Guangzhou, Guangdong, People's Republic of China; 2 Department of Orthopedics, Renmin Hospital of Wuhan University, Wuhan, Hubei, People's Republic of China; Pennington Biomedical Research Center, United States of America

## Abstract

Osteoarthritis, also known as degenerative arthritis or degenerative joint disease, causes pain and disability worldwide. Cartilage regeneration is key to finding a cure for this disease. Adipose-derived stem cells (ASCs) are capable of differentiating into cartilage lineages in vitro and they have shown promise in the field of regenerative medicine. However, the underlying mechanisms remain unclear. In this study, we demonstrated that miR-194 levels gradually decreased during the chondrogenic differentiation of human ASCs (hASCs). After predicting the target of miR-194 using Pictar and Targetscan, we hypothesized that Sox5 is potentially the key link between miR-194 and the chondrogenesis of ASCs. Initially, we demonstrated that Sox5 is a target of miR194 according to luciferase assay analysis. We further demonstrated that the differentiation of ASCs can be controlled by miR-194 through gain or loss of function experiments, and we observed that the down-regulation of miR-194 increases its direct target gene, Sox5, and results in enhanced chondrogenic differentiation of hASCs, whereas up-regulation decreases Sox5 and inhibits chondrogenesis. We also found that miR-194 correlates with Sox5 in osteoarthritis. These findings, taken together, are the first to illustrate the critical role of miR-194 in hASC chondrogenesis, and may provide novel insight beneficial to cell manipulation methods during cartilage regeneration.

## Introduction

Age-related osteoarthritis (OA) is widely recognized as the most common form of arthritis and one of the leading causes of pain and disability worldwide. OA refers to a cluster of clinical syndromes that include joint pain, swelling, functional limitation, and reduced quality of life [1]. Adipose-derived stem cells (ASCs) have shown promise in the field of regenerative medicine and are reported to exhibit stable proliferation kinetics and to exhibit the capacity to differentiate into various lineages *in vitro* [2]. Compared to bone marrow, which is widely used to isolate bone marrow-derived stem cells (MDSCs), adipose tissue holds the advantage of being easily obtained and being conducive for the separation of stem cells from the harvested tissue [3]. The ability to successfully direct ASCs toward cartilage lineage differentiation provides considerable progress toward the regeneration of articular cartilage [4]. Therefore, elucidation of the mechanisms underlying cartilage differentiation from adult stem cells may potentially prevent further cartilage destruction and activate the repair of cartilage lesions.

Chondrogenic differentiation is regulated by several transcription factors and growth factors. Various TGFβ proteins have been previously demonstrated to promote chondrogenesis due to their effects on induction of fibronectin, N-CAM, N-Cadherin, or tenascin, which are required for prechondrogenic cell aggregation [5], [6]. Many members of the family of bone morphogenetic proteins (BMPs) have been shown to promote chondrogenesis. The combination of BMP-4 and BMP-3 stimulates chondrogenesis in limb bud mesodermal cells [7], BMP-2, −4, and −7 coordinate to regulate the patterning of limb elements, and the effect is dependent upon differential expression of BMP receptors and BMP antagonists, such as noggin and chordin, respectively [8]. The members of the SRY-related family of transcription factor Sox play multiple roles during development. The expression of Sox9 is the earliest sign of commitment to chondrogenic differentiation of embryonic undifferentiated mesenchymal cells, and it is expressed in all chondroprogenitors and chondrocytes, but not in the more greatly differentiated hyperthrophic chondrocytes [9]. In contrast, Sox5 and Sox 6 are expressed in the differentiating cartilage elements rather than in prechondrogenic aggregates, and control the production of the cartilage extracellular matrix [10], [11].

Mature microRNAs (miRNAs) are 21–23-nucleotide (nt) noncoding RNAs that regulate post-transcriptional gene expression in various species [12]. After transcription, miRNA precursors undergo primary cleavage by Drosha in the nucleus, and are then exported to the cytoplasm by exportin where they udergo secondary cleavage by Dicer, and are subsequently inserted into RNA-induced silencing complex (RISC) [13], [14], [15], [16]. MiRNAs bind to the 3′-untranslated region (3′-UTR) of targeted mRNAs, which usually results in translational repression or target degradation, and, therefore, gene silencing. Accumulating evidence suggests that miRNAs play critical roles in the regulation of cellular processes such as proliferation, differentiation, and apoptosis [17]. MiRNAs are also reported to play roles in controlling chondrogenic differentiation. MiR-140 is cartilage tissue-specific during embryonic development and it plays an important role in cartilage development via regulation of histone deacetylase 4 (HDAC4) [18]. Lin et al demonstrated that miR-199a* was capable of regulating chondrogenic differentiation and that the effect was potentially due to its target gene Smad1 [19]. Yang et al found that miR-145 was down-regulated during the chondrogenic differentiation of MSCs, and that it potentially influenced chondrogenic differentiation due to suppression of Sox9 expression [20]. However, there is little evidence available that demonstrates the participation of miRNAs in the regulation of ASCs chondrogenic differentiation.

In this study, we found that miR-194 levels gradually decreased during chondrogenic differentiation of ASCs. We demonstrated that miR-194 targets and suppresses the expression of SRY-related high mobility group-Box gene 5 (Sox5). We further demonstrated that differentiation of ASCs can be controlled by miR-194 through gain- or loss-of-function experiments. We also found that miR-194 and Sox5 play a role in osteoarthritis. Our results suggest that miR-194 is a key mediator during chondrogenic differentiation via elimination of the effect of transcription factor Sox5.

## Materials and Methods

### Cell culture

The human ASCs were purchased from Cyagen Biosciences Technology, the standard characterization assay (including osteogenic, adipognic and chondrogenic differentiation assay) were all performed by Cyagen as quality control. The ASCs were cultured in hASC complete growth medium (Cyagen, Guangzhou, Guangdong, China). When the cells had grown in culture flasks to 70%–80% confluence, they were detached with trypsin. After centrifugation, cells were plated at approximately 6000 cells/cm^2^. At passage five, the cells were used to assess differentiation and determination of their biological identity. The HEK293 cells were purchased from Shanghai Institutes for Biological Sciences of the Chinese Academy of Sciences, and they were cultured in DMEM/F12 supplemented with 10% fetal bovine serum. All cells were cultured in a 37°C, humidified incubator with a mixture of 95% air and 5% CO2.

### Animals and animal care

A total of 10 Wistar male rats about 2 weeks old, weighing 130–150 g were purchased from the Experimental Animal Center of Wuhan University, China. The care and use of animals followed the recommendations and guidelines of the National Institutes of Health, and was approved by the Wuhan University Animal Care and Use Committee.

### Primary culture of cartilage cells

Articular chondrocytes were isolated from the knee joints of Juvenile rats as described previously [21]. In brief, cartilage tissues were cut into small pieces and digested with 0.25% trypsin and 0.1% type II collagenase for 30 min and 2 hours, respectively. The released cells were cultured in DMEM/F12 medium supplemented with 10% FBS and antibiotics. Cell viability was determined using typan blue stain. After the cells has reached confluence, IL-1β(10 ng/mL) was added to the culture medium, DMSO were used as controls.All experiments were repeated three times.

### RNA isolation and microarray

Total RNA was isolated using Trizol reagent (Invitrogen, Eugene, OR, USA) according to the manufacturer's instructions. Small RNAs were size-fractionated (<300 nt) by YM-100 Microcon centrifugal filter (Millipore, Billerica, MA, USA). RNA quality control procedures, labeling, hybridization, and scanning were performed by LC Sciences. The array sequence contents were derived from the Sanger Institute miRBase release 17.0 using the latest probe content in the Sanger miRBase.

### Luciferase Assays

The luciferase reporter was constructed based on the pMIR-REPORT vector (Ambion, Austin, TX,USA). The sense and anti-sense strands of the oligonucleotides of the Sox5-3′-UTR containing the miR-194 binding site were synthesized, annealed, and then subcloned into the pMIR-REPORT vector. The scrambled sequences were subcloned into the same vector in order to act as negative control. The Sox5-3′-UTR-miR194 or control plasmid was transfected into the HEK293 stable cell line using Lipofectamine 2000 (Invitrogen, Eugene, OR, USA). phRL-TK Renilla luciferase plasmid (Promega, Madison, WI, USA) was cotransfected for normalization of transfection efficiency. Measurements of luminescence were performed using the luminometer.

### Transfection assay

Next, we further analysed the functional relevance of miR-194, Pre-miR-194 and anti-miR-194 were designed as described previously [22]. Scrambled sequences of RNA duplex which was nonhomologous to any human genome sequences was act as negative control. 2′-O-methyl analogs were used to enhance RNA stability. We transfected the pre-miR-194, anti-miR-194, or their negative controls into hASCs in 6-well plates (10^5^ cell per well) with 5 ul siPORT NeoFX transfection agent (Ambion, Austin, TX,USA) following the manufacturer's instructions. After incubation for 24 h, the transfected cells were trypsinized and subjected to luciferase assay or the chondrogenic differentiation assay. After the indicated time points, the cells were harvested for mRNA and protein analysis.

### Chondrogenic Differentiation Assay

The hASCs prepared for miR-194 expression analysis were directly induced to chondrogenic differentiation. For gain- or loss-of-function analysis, hASCs were transfected with pre-miR-194, anti-miR-194, or their negative controls before chondrogenic differentiation and after transfection and incubation for 24 h. High density micromass regions were used for chondrogenic differentiation culture. The cells were trypsinized by trypsin and adjusted to a density of 10^7^ cells/mL. Next, 10 ul of the suspension was placed into the center of each well on a 12-well plate. After incubation for 2 h, wells were flooded with 1 mL chondrogenic differentiation medium (Cyagen, Guangzhou, Guangdong, China). The chondrogenic differentiation medium is composed of dexamethasone, ascorbate, ITS+ Supplement, sodium pyruvate, proline and TGF-β3, it was replaced every two days.

### Quantitative RT-PCR Analysis

Total RNA containing small RNA was extracted from the cell lines using TRIzol reagent (Invitrogen, Eugene, OR, USA) according to the manufacturer's instructions. We performed qRT-PCR to detect miR expression using the TaqMan miR assay kits (Ambion, Austin, TX,USA) according to the manufacturer's protocol. Levels of miR were normalized using rRNA U6 as a reference. To determine the expression levels of Sox5, Col2a1, Col9a2, Col11a1, Agc1 and COMP at the different stages of chondrogenic differentiation, cDNA was synthesized from total RNA using RT-PCR detection kit according to manufacturer's instructions (Epicentre Biotechnologies, Madison, WI, USA), and subsequently the synthesized cDNA was analyzed by qPCR using qPCR System (Promega, Madison, WI, USA) with SYBR Green (Invitrogen, Eugene, OR, USA) in which β-actin acted as an internal control, primers are listed in Table 1. Data was analyzed using the 2-ΔΔCt method. All experiments were performed in triplicate.

**Table 1:** Sequences of primers for qRT-PCR.

Gene name		Primer sequences
Sox5	Forward	5′-GGTGGCTGCTGTGACAAAGGGA-3′
	Reverse	5′-ACGGAGAGGCTGGTCGCTTG-3′
Col2a1	Forward	5′-CGCCGCTGTCCTTCGGTGTC-3′
	Reverse	5′-AGGGCTCCGGCTTCCACACAT-3′
Col9a2	Forward	5′-GCGCTATCGGTGCCACTGGG-3′
	Reverse	5′-GGGGGCCCGTTGCTCCTTTC-3′
Col11a1	Forward	5′-AAACCGGCCCAGTCGGTCCT-3′
	Reverse	5′-CAGGACCAGGGATGCCCCGA-3′
Agc1	Forward	5′-TGAGAACTGGCGCCCCAACC-3′
	Reverse	5′-AGGTGGTGGCTGTGCCCTTTT-3′
COMP	Forward	5′-CACCGCCTGCGTTCTTCTGCT-3′
	Reverse	5′-CCCAGGTCTGAGCCCAACGG-3′
β-actin	Forward	5′-TGGCACCCAGCACAATGAA-3′
	Reverse	5′-CTAAGTCATAGTCCGCCTAGAAGCA-3′

### Western Blot analysis

The cell lysates from the micromass cultures of hASCs transfected with pre-miR-194, anti-miR-194, or their negative control were extracted with lysis buffer according to the manufacturer's instructions (Sigma, St. Louis, MO, USA). After we measured the protein concentration, the equal protein samples were mixed with 5× sample buffer and boiled. The samples were resolved by 10% SDS-PAGE gel and transferred onto the PVDF membrane (Millipore, Billerica, MA, USA) using the semi-dry transfer method. After blocking in 10% nonfat dried milk in TBST for 2 h, the blots were incubated with anti-Sox5(1∶700 from rabbit; sc-20091, Santa Cruz Biotechnology, Inc., Santa Cruz, CA, USA) or anti-β-actin (1∶1000 from rabbit; sc-1616-R, Santa Cruz Biotechnology, Inc., Santa Cruz, CA, USA) at 4°C overnight. β-actin acted as an internal control. After washing with TBST, the blots were incubated with a horseradish peroxidase-conjugated secondary antibody (1∶2000 from goat; sc-2004, Santa Cruz Biotechnology, Inc., Santa Cruz, CA, USA) at room temperature for 1 h. After washing with TBST, enhanced chemiluminescence was used for detection, and they were developed using X-ray film.

### Statistical Analysis

Data are expressed as the mean±SD. Statistical comparisons were made between two groups with the t-test and between multiple groups with one-way ANOVA. A value of *P*<0.05 was considered to represent a significant result unless otherwise described.

## Results

### Differential Expression of miR-194 during hASCs chondrogenic differentiation

**Figure 1:**
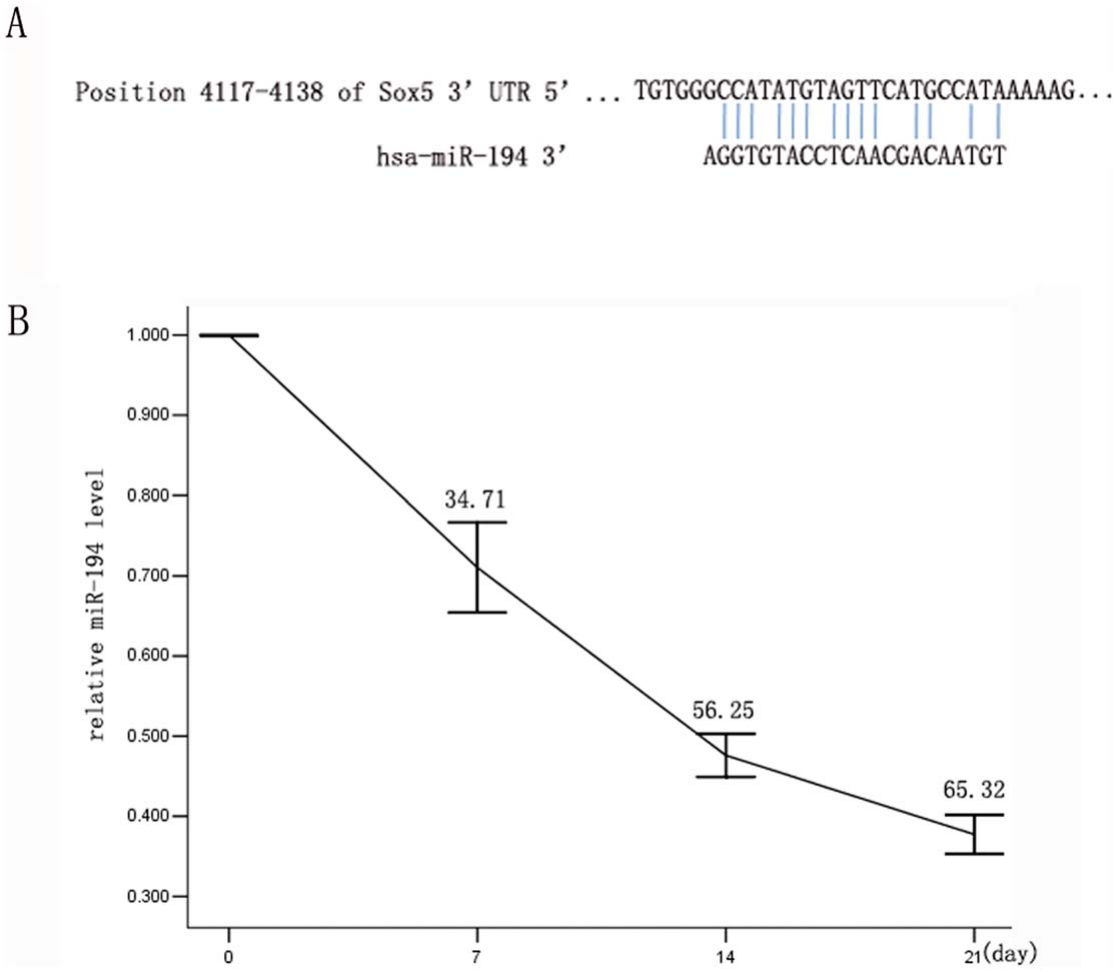
Differential expression of miR-194. (A) Bioinformatics study shows that miR-194 sequence is partially complementary to the 3′-UTR of Sox5 mRNA. (B) Differential expression of miR-194 during chondrogenic differentiation of hASCs (7 d, 14 d, 21 d). The relative gene expression alteration was demonstrated by percentage decrease. The relative expression level of miR-194 in untreated hASCs (0 d) was acted as control.

We performed miR microarray to detect the expression profile of miRs at three different stages during chondrogenic differentiation, including induction at 7 d, 14 d, and 21 d. We found that the expression of miR-194 significantly decreased during chondrogenic differentiation. Subsequently, we predicted the target genes of miR-194 using Pictar and Targetscan [23], [24] (Fig. 1A). We observed that Sox5 was clearly the potential target gene regulated by miR-194. Because of the principal role played by Sox5 in the process of MDSC differentiation into chondrocytes, we hypothesized that Sox5 may be inhibited by miR-194, which prevents ASCs from differentiating into chondrocytes. Furthermore, down-regulation of miR-194 potentially exhibits a positive effect on chondrogenic differentiation. Thus, we performed the qRT-PCR assay to validate the expression pattern of miR-194. The results confirmed that expression levels of miR-194 gradually decreased during ASC chondrogenesis (Fig. 1B).

### Sox5 is a target of MiR-194

**Figure 2:**
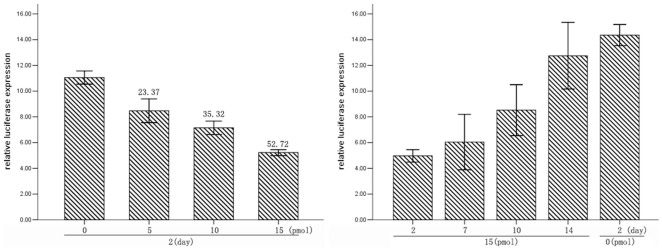
Sox5 is a target of miR-194. (A) Different doses of pre-miR-194 were co-transfected with the specific pMIR-REPORT construct into HEK293 cells. All cells were harvested at 48 h after transfection, and then luciferase activities were measured and normalized to the phRL-TK activities. The relative gene expression alteration was demonstrated by percentage decrease. Three independent transfection experiments were done. (B) The effect of pre-miR-194 gradually diminished in two weeks.

MiRNAs inhibit mRNA expression by binding the 3′-UTR of target mRNA. The Sox5 3′-UTR contains one putative miR-194 binding site which is bound with imperfect complementation. To determine if miR-194 targets Sox5, we applied the luciferase reporter gene assay using the pMIRREPORT Luciferase reporter. We co-transfected the Sox5-3′-UTR-miR194 reporter plasmid into HEK293 cells with pre-miR-194 or its control pre-miR, and we found that luciferase activity was significantly decreased in the HEK293 cells transfected with pre-miR-194, and that the effect was dose-dependent (Fig. 2A). These data indicate that miR-194 can suppress expression of transcripts containing a miR-194-binding site according to our luciferase reporter assay analysis. We also determined the duration of our transfected oligoribonucleotides. We found that the effect gradually diminished, and there was no significant difference between transfected and control group two weeks post-transfection.

### MiR-194 inhibits Sox5 expression during chondrogenic differentiation

**Figure 3:**
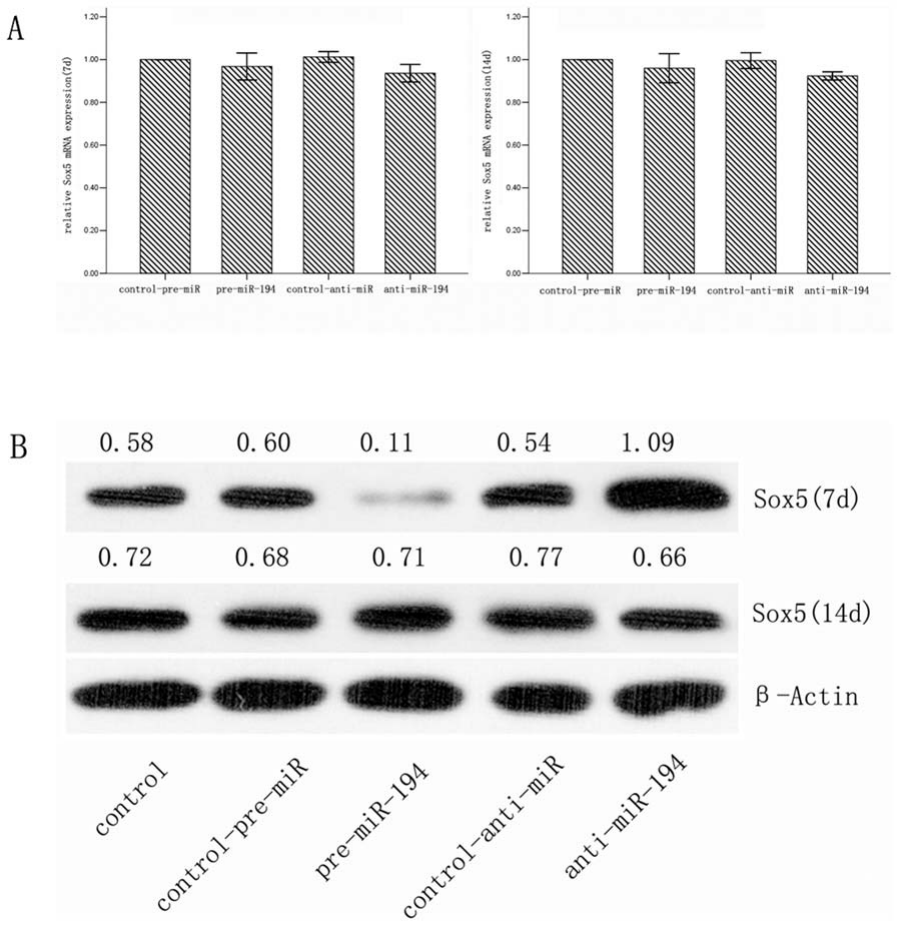
MiR-194 represses the expression of Sox5 at protein level during chondrogenic differentiation. (A) hASCs were transfected with pre-miR-194, anti-miR-194 or their control respectively. After induction for 7 d and 14 days, the cells were harvested for measurement of Sox5 mRNA using qRT-PCR (B) and the protein expression level using Western blot. Untreated cells was set as control, andβ-actin acts as an internal control.

To demonstrate whether miR-194 acts as an inhibitor of Sox5 protein expression, we transfected hASCs with pre-miR-194, anti-miR-194, and their negative control for 24 h, respectively, and then exposed the transfected cells to the chondrogenic differentiation medium that primarily consisted of TGF-β3. We detected the expression of Sox5 using qRT-PCR and western blot assay. Compared to the negative control, we found that there was no significant change on the mRNA level of Sox5 (Fig. 3A), while in contrast, its protein level was notably decreased in the pre-miR-194 group on day 7, and it was increased in the anti-miR-194 group (Fig. 3B). These results indicated that miR-194 potentially represses the protein expression whereas the mRNA level remains undisturbed. However, the difference was decreased at day14, which is likely due to the degradation of transfected oligoribonucleotides.

### MiR-194 inhibits early chondrogenic differentiation

**Figure 4:**
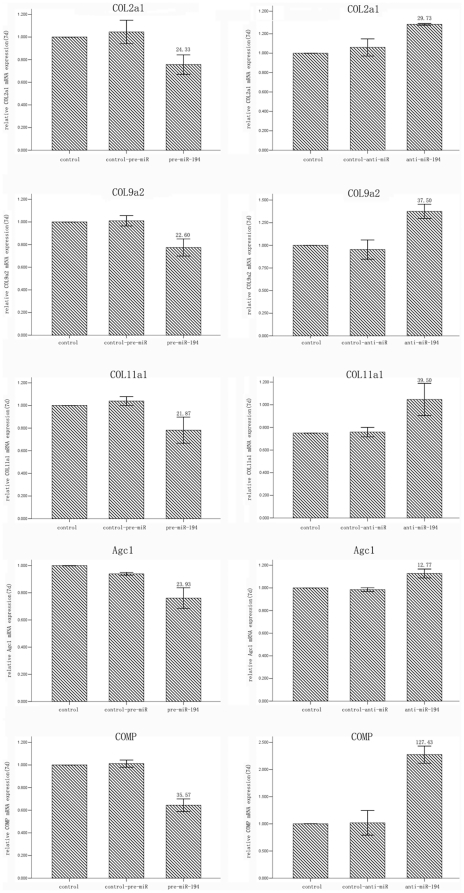
miR-194 regulates chondrogenic differentiation of hASCs. (A) hASCs were transfected with pre-miR-194 or their control respectively. (B) hASCs were transfected with anti-miR-194 or their control respectively. After 7 days of induction, all the cells were lysed and measured the expression of chondrogenic differentiation markers via qRT-PCR. The relative gene expression alteration was demonstrated by percentage decrease or increase. Untreated cells was set as control, and three independent cell culture experiments were done.

We further explored whether miR-194 exhibited an effect on chondrogenic differentiation: we transfected pre-miR-194, anti-miR-194, and their negative control into hASCs, we used 15 pmol in all experiments. Total RNA was extracted from the micromass after induction of chondrogenic differentiation for 7 d and 14 d, and qRT-PCR analysis was applied to detect the mRNA level of the chondrogenesis markers, which included Col2a1, Agc1, COMP, Col9a2 and Col11a1. Compared to the negative control group, hASCs transfected with pre-miR-194 demonstrated a significant decrease in the mRNA expression levels of chondrogenesis markers, whereas inhibition of endogenous miR-194 expression in hASCs by transfection of anti-miR-194, under the same induction conditions as above, resulted in enhanced chondrogenic differentiation as demonstrated by the significant increase in chondrogenesis markers at the mRNA level (Fig. 4). These results revealed that modulation of miR-194 affected the expression of genes related to chondrocytes after induction for 7 d and 14 d. However, the difference decreased at day14 (data not shown).

### MiR-194 correlates with Sox5 in IL-1β induced osteoarthritis

**Figure 5:**
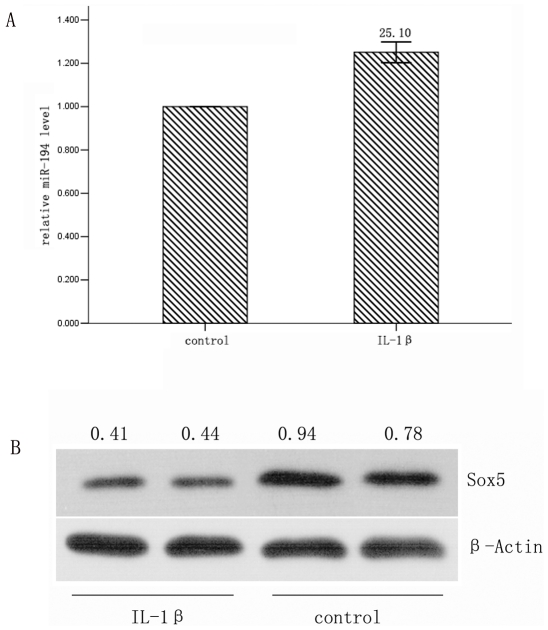
miR-194 is in accordance with Sox5 in osteoarthritis. (A) Primary chondrocyte was treated by IL-1βfor 24 h, DMSO was added as control group, qRT-PCR showed that miR-194 was up-regulated, The relative gene expression alteration was demonstrated by percentage increase and (B)Western-blotting Assay showed that Sox5 was down-regulated compared with control group. Three independent cell culture experiments were done.

We further asked whether the expression of miR-194 is in accordance with Sox5 in osteoarthritis, we treated primary chondrocyte with IL-1βfor 24 h and the total RNA and protein was collected for qRT-PCR and Western-blotting Assay. We found that miR-194 was up-regulated in the osteoarthritis group while Sox5 was down-regulated compared with control group (Fig. 5).

## Discussion

Currently there are no disease-modifying therapeutics available for patients suffering from OA. Therapeutic options are limited to oral and intra-articularly injected pain-relief medications, and joint replacement surgery. Patients with OA present with joints that exhibit a damaged matrix in which repair is insufficient, this implies that key factors are missing and, thus, regenerative processes do not function properly. Molecules that activate the chondrogenic potential of adult stem cells may potentially prevent further cartilage destruction and stimulate repair of cartilage lesions.

MiRs recognize their target(s) with partially/completely complementary sequences and repress their translation or modify their stability. MiRs play essential roles in stem cell maintenance and differentiation [25], [26], [27]. Our study provides new insight into the precise mechanisms underlying the control of cell fate decisions. We postulated that the miRNAs inhibited during chondrogenesis of ASCs potentially play a role in chondrogenesis. Using miR microarray analysis, we demonstrated that miR-194 gradually decreased during chondrogenesis of ASCs, the differential expression pattern was confirmed by qRT-PCR assay. We demonstrated the predicted target proteins for miR-194 using bioinformatic approaches, we found that one of the target proteins was Sox-5. Because Sox5 plays an essential role in chondrogenesis, we focused on the relationships among miR-194, Sox5, and chondrogenesis. In this study, our findings indicated that miR-194 suppresses the chondrogenic differentiation of ASCs by directly targeting Sox5, a key transcriptional factor for chondrogenesis at the post-transcriptional level. Following induction, the decrease in expression of miR-194 allows for the positive effect on chondrogenesis of Sox5, thereby facilitating chondrogenic differentiation.

MiR-194 is thought to regulate cell differentiation in the gastrointestinal tract. Previous reports have shown that miR-194 is highly up-regulated with great tissue specificity during intestinal epithelial differentiation, and that the expression pattern is controlled by HNF-1a [28]. It has also been reported that miR-194 is down-regulated in hepatic stellate cells during liver fibrogenesis, and that over-expression of miR-194 causes decreased stellate cell activation [29]. MiR-194 is also reported to participate in carcinogenesis. Meng et al found that miR-194 is a hepatocyte-specific marker in the liver and plays a role in EMT and HCC metastasis [30]. Dong et al suggested that miR-194 inhibits epithelial to mesenchymal transition (EMT) of endometrial cancer cells by targeting oncogene BMI-1 [31]. Beyond the findings of these reports, we have identified a novel function concerning the inhibition of Sox5 mediated chondrogenesis.

The members of the SRY-related family of transcription factor Sox include over 30 members in vertebrates characterized by a DNA-binding domain known as the high-mobility group (HMG) box. They play multiple roles in development, among which Sox9, Sox5, and Sox6 are the three most important that participate in chondrogenesis. Sox5 belongs to a different subgroup of Sox proteins, it exhibits a high degree of sequence identity with Sox6 and no sequence homology with Sox9 [32]. In contrast to Sox9, which is largely expressed in prechondrogenic cells, Sox5 together with Sox6 is expressed in differentiating cartilage cells, and they activate Col2a1 and aggrecan genes in coordination with Sox9 in vitro, and therefore play an important part in controlling the extracellular matrix production [10], [33]. The physiological roles of Sox5 in chondrogenic differentiation were demonstrated by genetically manipulated mice in which a single deletion of Sox5, using homozygous Sox5 mutant (Sox5−/−) mice, demonstrated relatively mild skeletal anomalies, whereas a double deletion of both the Sox5 and Sox6 genes using Sox5−/−Sox6−/− mice has resulted in severe alterations in cartilage differentiation [34].

The luciferase reporter analysis demonstrated that exogenous pre-miR-194 and anti-miR194 regulated the activity of luciferase when the miR-194 miRs regulatory element (MRE) from Sox5 3′-UTR was fused to luciferase, and that suppression was exhibited in a dose-dependent manner. These results suggest that miR-194 potentially regulates Sox5 expression by binding the MREs within the Sox5 3′-UTR, and prompted us to investigate whether miR-194 affects chondrogenesis via targeting of Sox5. Consistent with the prominent role of Sox5 in the regulation of chondrogenesis, our results demonstrated that over-expression of miR-194 via pre-miR-194 precursor resulted in inhibition of hASCs chondrogenic differentiation, whereas inhibition of miR-194 enhanced chondrogenic differentiation.

MiRs inhibit targeted gene expression through two distinct pathways, which are dependent upon whether the miRs and the target mRNA are complementary. MiRs suppress mRNA translation with imperfect complementary target sequences, and degrade mRNA bearing perfect complementary target sequences [35], [36]. Computational algorithms predicted that miR-194 binds to Sox5 3′-UTR with imperfect complementation, suggesting that it potentially does not degrade Sox5 mRNA. Consistent with the mechanisms of miRs regulation, both in gain- or loss-of function of miR-194 experiments, we found Sox5 differentially expressed at the protein level but not at the mRNA level. This effect vanished at the 14^th^ day, potentially due to the degradation of the plasmids and subsequent low transfection efficiency. This study revealed that miR-194 repressed Sox5 expression via its binding with imperfect complementation to the Sox5 mRNA 3′-UTR through translational inhibition.

What is more important, we found that MiR-194 was elevated in IL-1β induced osteoarthritis, and it is accompanied by decreased expression of Sox5. The results implied that MiR-194 and Sox5 were deregulated during osteoarthritis, and they are likely to be target for osteoarthritis therapy.

Besides Sox5, there are many additional proteins that seem to be regulated by MiR-194 using TargetScan, among which we found that Sox6 is also a target for MiR-194. Sox6 is also a critical factor in chondrogenesis. This result again proved our hypothesis that MiR-194 regulates chondrogenesis. Sox9 is reported to be an early determinant during chondrogenesis, however, it is not predicted to be one of MiR-194's targets, so the results implied that MiR-194 might play important role during late chondrogenesis.

In conclusion, our study demonstrates that miR-194 is decreased during chondrogenic differentiation of hASCs. The down-regulation of miR-194 increases its direct targeting of gene Sox5, and results in enhanced chondrogenic differentiation of hASCs, what is more, we found that MiR-194 was up-regulated and Sox5 was down-regulated in osteoarthritis. To our knowledge, this is the first study to demonstrate the critical role of miR-194 in hASC chondrogenesis and osteoarthritis, and it may provide novel insight toward potential cell manipulation improvements during cartilage regeneration. However, an in vivo study and the relationships in human is still need to be illustrated.
